# Comparison of the therapeutic effects of 15 mg and 30 mg initial dosage of prednisolone daily in patients with subacute thyroiditis: protocol for a multicenter, randomized, open, parallel control study

**DOI:** 10.1186/s13063-020-04337-8

**Published:** 2020-05-24

**Authors:** Shaoyong Xu, Yuxin Jiang, Aihua Jia, Juan Zhang, Bin Gao, Jing Xu, Xiaorui Jing, Yang Jiao, Jia Wei, Wenlei Xu, Ruikun Chen, Ling Gao, Lei Shang

**Affiliations:** 1 Department of Health Statistics, Shaanxi Key Laboratory of Free Radical Biology and Medicine and the Ministry of Education Key Lab of Hazard Assessment and Control in Special Operational Environment, School of Public Health, Air Force Medical University, Changle West Road No. 169, Xi’an, Shaanxi 710032 China; 2 Department of Endocrinology, Xiangyang Central Hospital, Affiliated Hospital of Hubei University of Arts and Science, No. 136, Jingzhou Street, Xiangyang, Hubei 441021 China; 3 Department of Clinical Medicine, Medical College of Yan’an University, No. 38, Guanghua Road, Yan’an, 716000 China; 4 Department of Endocrinology, No.1 Hospital of Yulin, Yulin, 719000 China; 5 Department of Endocrinology, 3201 Hospital of Xi’an Jiao tong University Health Science Center, 783 Tianhan Road, Hanzhong, Shaanxi 723000 China; 6 Department of Endocrinology, Tangdu Hospital, Air Force Medical University, Changle West Road No. 169, Xi’an, Shaanxi 710032 China; 7 Department of Endocrinology, The Second Affiliated Hospital of Xi’an Jiaotong University, Xi’an, 710032 China

**Keywords:** Subacute thyroiditis, Prednisolone, Randomized controlled trial, Chinese population

## Abstract

**Background:**

Subacute thyroiditis (SAT) is the most common cause of thyroid pain. Several clinical guidelines recommend that patients who fail to respond to full doses of non-steroidal anti-inflammatory drugs (NSAIDs) should be treated instead with oral corticosteroid therapy. However, albeit strong recommendations, the treatment protocol is based on low-quality evidence and high-quality clinical trials are lacking with respect to the optimal initiation dosage and usage of corticosteroid. We aimed to evaluate whether 15 mg/day of prednisolone (PSL) as the initial dosage could provide non-inferiority effectiveness but with lower risk and more safety compared with 30 mg/day of PSL as the initial dosage.

**Methods/design:**

This is a multicenter, open-label, randomized, parallel trial that will be conducted at five academic hospitals in China. A total of 90 adult patients diagnosed with SAT who present moderate to severe pain or fail to respond to full doses of NSAIDs will be randomly assigned with a 1:1 ratio to the low initial PSL dosage group (15 mg daily) and standard initial PSL dosage group (30 mg daily). The primary endpoint is the time period (days) required for PSL treatment (including PSL treatment for recurrence).

**Discussion:**

Our randomized controlled trial will try to determine the optimal protocol in the treatment of SAT by providing high-quality evidence.

**Trials registration:**

Chinese Clinical Trial Register, ChiCTR1900023884. Registered on 15 June 2019.

## Background

Subacute thyroiditis (SAT), also named subacute granulomatous or subacute painful thyroiditis or de Quervain thyroiditis, is a transient inflammatory thyroid disease [1]. SAT is the most common painful thyroid disease and the prevalence of SAT is nearly 5% in the population with abnormal thyroid function [2]. According to the data from the Rochester Epidemiology Project in Olmsted County, Minnesota, the incidence of SAT was reported as 12.1 cases per 100,000/year with a higher incidence in females than in males (19.1 and 4.1 per 100,000/year, respectively) [3, 4]. Over 95% of people with SAT often present with moderate-to-severe pain [3], which may be limited to the region of thyroid or radiate to the upper neck, jaw, throat, upper chest, or ears. The pain may begin focally and spread from one side to the other of the gland over several weeks. Patients may also have fever, fatigue, malaise, anorexia, and myalgia.

SAT is a self-limiting disease and the primary treatment is to relieve thyroid pain. Several clinical guidelines, such as the American Thyroid Association (ATA) [5] and the Chinese Endocrinology Association [6], recommend anti-inflammatory agents based on the severity of symptoms. Non-steroidal anti-inflammatory drugs (NSAIDs) are initially used, particularly for mild cases. Oral glucocorticoids (prednisolone [PSL]) are suggested when patients fail to respond, or present initially with moderate to severe pain and/or thyrotoxic symptoms. ATA and UpToDate Clinical Consultants recommend that patients who fail to respond to full doses of NSAIDs over several days (2–3 days) should be treated instead with oral corticosteroid therapy. PSL of 40 mg daily for 1–2 weeks followed by a gradual taper over 2–4 weeks or longer is the most popular recommendation [5]. According to the Chinese Endocrinology Association, the initial PSL dosage of 20–40 mg daily is recommended [6].

However, albeit strong recommendations, the treatment protocol is based on low-quality evidence [3, 7–11] and high-quality clinical trials are lacking with respect to the optimal initiation dosage and usage of corticosteroids. Considering the short- and long-term adverse effects on many organ systems and that the risk is both dose- and duration-dependent [12–16], it is essential to consider the possibility of starting with a minimal dosage of corticosteroid and stop for a short duration. A recent observational study showed that the treatment protocol of PSL 15 mg daily as the initial dosage with tapering by 5 mg every 2 weeks was effective and safe for Japanese patients [17]. In addition, a retrospective study found that PSL treatment with mean dose of 15.0 mg/day (range = 14–16 mg/day) was superior to NSAIDs with regard to resolution of symptoms [18]. Based on the findings, we hypothesized that a low initial dosage of PSL seemed effective in the treatment of SAT; unfortunately there was a lack of high-quality well-designed clinical trials.

Therefore, in the present randomized controlled trial (RCT), we will use 15 mg/day of PSL as the initial dosage for treatment in patients with SAT who fail to respond to full doses of NSAIDs or present initially with moderate to severe pain, with the aim of evaluating whether 15 mg/day of PSL could provide non-inferiority effectiveness but with lower risk and more safety compared with 30 mg/day of PSL.

## Methods

### Study design

This is a multicenter, open-label, randomized, parallel non-inferiority trial. The study will be conducted at five academic hospitals in China: (1) Xiangyang Central Hospital, Affiliated Hospital of Hubei University of Arts and Science; (2) No. 1 Hospital of Yulin; (3) 3201 Hospital of Xi’an Jiao tong University Health Science Center; (4) Tangdu Hospital, Air Force Medical University; and (5) The Second Affiliated Hospital of Xi’an Jiaotong University.

**Fig. 1:**
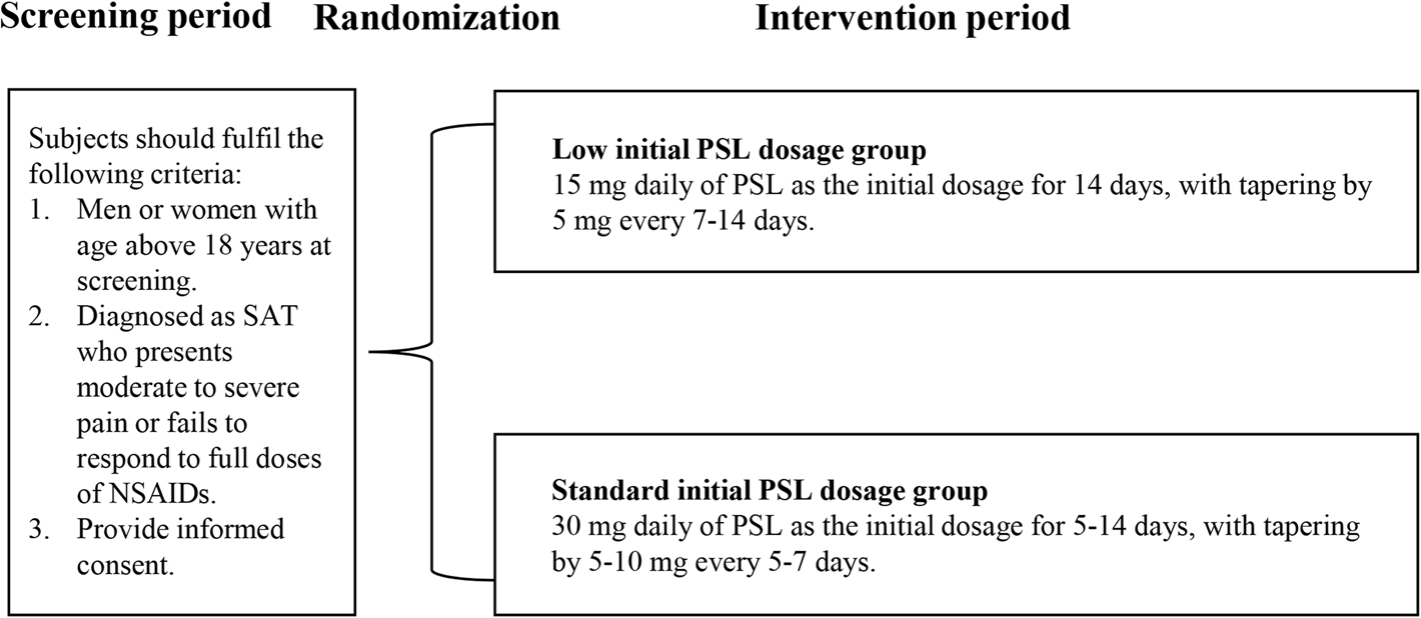
Study flow chart. SAT subacute thyroiditis, PSL prednisolone

Participants who fulfill the eligibility criteria will be randomly assigned with a 1:1 ratio to any of the following two groups: group 1 = low initial PSL dosage; and group 2 = standard initial PSL dosage. In group 1, 15 mg daily of PSL as the initial dosage for 14 days will be used, followed by a gradual taper by 5 mg every 7–14 days. In group 2, 30 mg daily of PSL as the initial dosage for 5–14 days will be used, followed by a gradual taper of 5–10 mg every 5–7 days (Figure 1).

### Study population

Individuals should fulfil the following criteria:

1. Age ≥ 18 years at screening;
2. Diagnosis of SAT and presenting with moderate to severe pain (sore > 7/10) or failing to respond to full doses of NSAIDs;
3. Provide informed consent before any study-specific procedures and not involved in another clinical study in the previous 3 months.

### Exclusion criteria

Individuals should not enter the study if any of the following exclusion criteria are fulfilled:

1. Women who are pregnant, intending to become pregnant during the study period, currently lactating women, or women of childbearing potential not using highly effective, medically approved methods of birth control;
2. Suspicion or diagnosis of acute suppurative thyroiditis, Graves’ disease, Hashimoto thyroiditis, or thyroid carcinoma;
3. Glucocorticoids allergy or intolerance (e.g. systemic allergic reaction, induced asthma, bleeding, ulcer and perforation of stomach or intestine);
4. Previous treatment with PSLs in the last 6 months before screening;
5. Adrenocortical hyperfunction, uncontrolled hypertension, or diabetes;
6. Patients with clinically apparent liver disease characterized by any of the following:
  - ALT or AST > 3× upper limit of normal confirmed on two consecutive measurements (by local laboratory) within 4 weeks before the screening period;
  - Impaired excretory (e.g. hyperbilirubinemia) and/or synthetic function, or other conditions of decompensated liver disease such as coagulopathy, hepatic encephalopathy, hypoalbuminemia, ascites, or bleeding from esophageal varices;
  - Acute viral or active autoimmune, alcoholic, or other types of hepatitis.
7. Patients with moderate /severe renal impairment or end-stage renal disease (estimated glomerular filtration rate ≤ 60 mL/min calculated by using the abbreviated equation developed by the Modification of Diet in Renal Disease study with modification for the Chinese population) at screening or within 4 weeks before screening (by local laboratory);
8. Congestive heart failure defined as New York Heart Association class III or IV;
9. Significant cardiovascular history within the past 3 months before screening defined as myocardial infarction, coronary angioplasty or bypass graft(s), valvular disease or repair, unstable angina pectoris, transient ischemic attack, or cerebrovascular accident;
10. History of mental illness;
11. History of corneal ulcer;
12. History of gastrointestinal disease including gastroenterostomy, enterectomy, Roemheld syndrome, severe hernia, and intestinal obstruction;
13. Postoperative patients with unhealing wound;
14. Diagnosed and/or treated malignancy (except for basal cell skin cancer, in situ carcinoma of the cervix, or in situ prostate cancer) within the past 5 years;
15. History of organ transplant or acquired immunodeficiency syndrome;
16. History of alcohol abuse or illegal drug abuse within the past 12 months;
17. Potentially unreliable patients and those judged by the investigator to be unsuitable for the study.

### Randomization methods and concealment mechanism

The stratified block randomization method will be used. Participants will be stratified by research center and the appropriate segment lengths will be selected. Based on the number of seeds, a random coding table of 92 participants will be generated using the statistical software SAS8.2 PROC PLAN. The randomized numbers will be segmented, retained, and managed by the third party who will not be involved in the data collection (Lei Shang). The investigators in research centers will enroll the participants. When needed, the investigators will ask for numbers from the third party by telephone, using the order in which the participants will be treated, and the intervention scheme with that serial number will be provided.

### Methods for ensuring blinding

The aim of the present study is to explore the effects of different dosages of PSL and a definite blindness is deemed worthless for investigators or for the patients. Therefore, an open-label study is conducted in which only laboratory personnel and data analysts (the third party) will be blinded; unblinding will not occur.

### Screening period

In the screening period, patients’ data on demographical information, physical examination, current medical conditions, and history of concomitant diseases will be collected. Laboratory examination including hematology panel, urinalysis, liver function, kidney function, thyroid function, erythrocyte sedimentation rate (ESR) and C-reactive protein (CRP) will be conducted. Thyroid ultrasound and radioactive iodine uptake rate of thyroid (thyroid scintigraphy or fine-needle aspiration cytology as alternatives) will be performed. The thyroid pain will be evaluated based on visual analog scales (VAS) and thyroid tenderness will be categorized by no (–), mild (+), moderate (++), and severe (+++) degrees. Heart rates and temperatures will be monitored. Since eligible patients will be randomized within 1–2 days after screening, laboratory results from the screening period will be considered as baseline data and there will be no need to repeat. However, other data such as thyroid pain, heart rate, and blood pressure should be assessed after randomization and considered as baseline data.

### Treatment period

Patients who fulfill the eligible criteria will be randomly assigned with a 1:1 ratio to treatment groups and drugs will be dispensed accordingly.

Group 1 is the low initial PSL dosage, in which 15 mg daily of PSL will be provided as the initial dosage for 2 weeks; the dosage will then taper by 5 mg every 7–14 days over 2–4 weeks or longer. Group 2 is the standard initial PSL dosage group, in which 30 mg daily of PSL will be provided as the initial dosage. The dosage will begin to taper 3–7 days after the complete resolve of thyroid pain and then taper by 5–10 mg every 5–7 days over 2–4 weeks or longer. Because most patients will receive complete resolve of thyroid pain within 5–10 days, 30 mg daily of PSL will be provided for around 5–14 days in the standard initial PSL dosage group. However, in the low initial PSL dosage group, 15 mg daily of PSL will be provided for 14 days regardless of the time period of complete pain resolve.

According to the clinical experience and the results of the pre-experiment, the pain of most patients will disappear completely within 5–10 days after receiving PSL treatment, no matter the low initial dose or the standard initial dose. Therefore, if the test group still has pain at 2 weeks, consider termination, adjust to the standard initial dose for treatment and specify the reason (with the exception of the situation that the pain completely disappears but the symptoms recur subsequently). In addition, whether in the low initial dose or in the standard initial dose group, if the pain does not relieve significantly 3 days after PSL is given, it is necessary to re-evaluate the diagnosis of SAT, eliminate other causes (such as acute thyroiditis), and give corresponding treatment if confirmed; if other causes are excluded, the study can be considered to be terminated, and the low initial dose can be adjusted to the standard initial dose for treatment and the reasons recorded in detail; the standard initial dose can be increased to a higher dose (such as 40 mg/day) and the reason specified.

The laboratory parameters that will be measured to assess efficacy and safety at each visit are shown in Table 1. The patients will receive face-to-face daily visit within the first week in research centers; thyroid pain, temperature, and adverse events (AE) will be assessed at each visit. Time periods required for the relief and complete resolution of thyroid pain will be recorded. Blood samples will be taken for the measurement of CRP and ESR in the first week. The patients will then receive telephone visits every 7–10 days. During each telephone visit, thyroid pain, temperature, and AEs will be investigated, medicine usage will be directed and recorded. If patients complain of pain in the tapering period, the tapering of the dosage will be delayed. In some cases with severe pain, the dosage will even increased; the dosage will then be tapered until the thyroid pain disappears. If PSL is tapered to a minimum dosage (i.e. 5 mg) for several days (3–7 days) with no thyroid pain, discontinuation of PSL will be considered and patients will receive a face-to-face visit. During the visit, physical examination (e.g. blood pressure and body weight) will be conducted and a blood sample will be taken for the measurement of thyroid function, liver function, kidney function, glucose level, CRP, and ESR. In addition, AEs will be collected. If SAT recurs after discontinuation of PSL, PSL will be administered again. During the treatment period, anti-ulcer drugs will be administered to some patients with a history of intestinal ulcer or who feel abdominal discomfort during treatment. A hepatologist will be consulted for possible antiviral therapy in patients with inactive hepatitis.

**Table 1:** Study plan detailing the procedures

Study period	Screening	Intervention period				
**Visit number**	1	2	3*	4-x	x	x + n
Study interval	– 0–3 days	Randomization	1 week	Every 1 week	PSL discontinuation	Every 4 weeks
Visit window*			(±2 days)	(±2 days)	(±2 days)	(±7 days)
Telephone visit				√		
*Screening/Demography/Baseline*						
Written informed consent	√					
Inclusion / Exclusion criteria	√	√				
Demographics	√					
Physical examination, height, and weight	√					
Medical / Current conditions	√					
History of diabetes and complications	√					
*Intervention*						
Instruction of drugs		√	√	√	√	√
Thyroid pain (VAS)	√	√	√	√	√	
Thyroid tenderness	√	√	√	√	√	
Temperature monitor	√	√	√	√	√	
CRP and ESR	√		√		√	
Thyroid function	√				√	√
24 h I-131 uptake or thyroid scintigraphy	√					
Hematology panel	√				√	
Liver function	√				√	
Creatinine, uric acid	√				√	
Glucose	√				√	
AEs		√	√	√	√	√

*AE* adverse event, *CRP* C-reactive protein, *ESR* erythrocyte sedimentation rate, *VAS* visual analog scale

*The patients will receive face-to-face daily visit within the first week in research centers; thyroid pain, temperature, and AEs will be assessed at each visit

About 50% of patients have an initial thyrotoxic phase due to unregulated release of performed thyroid hormones from damaged thyroid follicular cells [3]. The thyrotoxic phase usually lasts 3–6 weeks, ending when the thyroid stores of preformed hormones are depleted. About 30% of patients subsequently enter a hypothyroid phase that can last up to 6 months. In the present study, anti-thyroid drugs will have no role and beta-blockers will be used as needed to control thyrotoxic symptoms. Levothyroxine will be employed when patients have a TSH level above 10 mIU/L or symptoms during the hypothyroid stage. In addition, although viral infection is regarded as the possible cause of SAT, antiviral medications will not be suggested in the study.

After the discontinuation of PSL, patients will be invited to participate in the following observational study in which a 3-month face-to-face visit will be planed. The details of the follow-up study will be described elsewhere.

### Outcomes

The primary endpoint is the time period (days) required for PSL treatment (including PSL treatment for recurrence).

Secondary outcomes include time periods (days) required for complete resolution of thyroid pain (defined as no thyroid pain and no tenderness), time periods required for the relief of thyroid pain (defined as at least 50% decline of VAS scores), percentage of relapse during treatment, and the total dosage of PSL. Safety outcomes include all the AEs, vital signs, and collection of clinical chemistry parameters.

### Statistical methods

The primary and secondary outcomes will be analyzed based on the full analysis set. All outcomes will also be analyzed based on the per-protocol set. The security endpoint will be analyzed based on the safety analysis set. Supportive sensitivity analysis will be carried out based on the per-protocol set and safety analysis set, using the method of last observation carried forward. Descriptive analysis will be conducted for continuous variables such as time period required for PSL treatment, total dosage of PSL, body weight, blood glucose, blood lipids, and blood pressure. Indexes such as the percentage of relapse will be summarized using frequencies and percentages. The AEs and their incidence will be summarized. The mid-term analysis will be carried out at the end of the PSL treatment, before the follow-up study.

### Sample size estimate

The present study is a non-inferiority trial. The primary endpoint is the time period (days) required for PSL treatment (including PSL treatment for recurrence). PASS 11.0 software (NCSS, LLC, Kaysville, UT, USA.) was used to estimate sample size.

According to the previous literature [17, 18], the mean time was around 36 days with a standard deviation of 12 days. With a one-sided alpha level of 0.05, group sample sizes of 38 and 38 will achieve the power of 81% to detect non-inferiority with the margin of 7 days and the standard deviation of 12 days. Considering a drop-out rate of 20%, a sample size of 90 patients with a 1:1 allocation rate (45 patients per group) was finally determined.

### Data management

Investigators will collect and record data on the case report form and input the data to the electric database. All data will be stored in the database and inspected by a third party who is not involved in this study. The doctors and statisticians will not have access to these data until patient evaluations are completed.

The steering committee consists of the principal investigators, the local investigators at participating study sites, the statisticians, and the trial coordinators. The steering committee remains responsible for the interpretation of the data and drafts the final report that will be approved by all investigators.

The Data Monitoring Committee (DMC) will comprise representatives from the School of Public Health, Air Force Medical University of PLA. The DMC will be formed independently from the funders. The DMC will be responsible for the independent assessment of the validity and integrity of the randomized parallel control study. The DMC will meet twice a year or more if needed. A monitor will conduct monitoring visits once every 3 months and the auditing trial conduct will be independent from investigators and the sponsor.

## Discussion

Corticosteroids are important in the treatment of subacute thyroiditis; however, numerous adverse effects have been attributed to corticosteroids. Several retrospective reports have shown that long-term use of corticosteroids, even in low doses, is a significant independent predictor of adverse effects [12–16]. For example, a study found that the average daily prednisone dose was the strongest predictor of an adverse effect potentially attributable to glucocorticoid therapy (odds ratio = 4.5 for 5–10 mg and 32.3 for 10–15 mg) [12]. Studies also suggest that even short-term use of glucocorticoids may be associated with serious adverse effects. For example, a retrospective cohort study included 327,452 adults aged < 65 who received at least one short-term prescription over a 3-year period; results showed that there was an increase in the rates of sepsis, venous thromboembolism, and fracture [16]. Adverse effects may be not necessarily serious but displeasing to patients (e.g. Cushingoid appearance), life-threatening (e.g. serious infections), symptomatic (e.g. gastrointestinal ulcer), or largely asymptomatic until a later manifestation develops that requires medical attention (e.g. acute vertebral collapse). However, due to the relative short-term study period in the present trial, some long-term AEs cannot be collected and we may have difficulty in comparing adverse effects between groups, which is the limitation of the study. Meanwhile, because the risk is both dose- and duration-dependent, the time period of PSL usage was chosen as the primary endpoint; if non-inferior results are obtained, a lower initial dosage of PSL with a non-inferior time period will theoretically indicate reduced adverse effects.

This article presents the rationale and design of a RCT to test the effectiveness and safety of 15 mg daily of PSL versus 30 mg daily of PSL as the initial dosage in patients with SAT. This is the first RCT on this topic in Chinese people; by providing high-quality evidence, the present study will try to address the question of whether a lower initial dosage of PSL could provide non-inferiority effectiveness but with lower risk and more safety. If confirmed, our clinical practice may be modified to some extent, and patients may ultimately benefit.

### Trial status

The trial is currently recruiting participants. The recruitment began in June 2019 and is anticipated to end in March 2021. Trial registration number: ChiCTR1900023884. Registration date: 15 June 2019. The name of the registry is Chinese Clinical Trial Registry and the URL of the trial registry record is https://www.chictr.org.cn/.

## Data Availability

The datasets used and/or analyzed during the current study are available from the corresponding author on reasonable request.

## Abbreviations

SAT: Subacute thyroiditis
NSAIDs: Non-steroidal anti-inflammatory drugs
PSL: Prednisolone
ATA: American Thyroid Association
ESR: Erythrocyte sedimentation rate
CRP: C-reactive protein
VAS: Visual analog scales
